# Effect of Anterioposterior Weight-Shift Training with Visual Biofeedback in Patients with Step Length Asymmetry after Subacute Stroke

**DOI:** 10.3390/jpm13121726

**Published:** 2023-12-18

**Authors:** Yea Jin Jo, Dae Hyun Kim, Seeun Kim, Jung Hoon Kim, Jong Hyun Choi, Jong Bum Park, Yoon Su Baek, Yoon Ghil Park, Deog Young Kim

**Affiliations:** 1 Research Institute of Rehabilitation Medicine, Yonsei University College of Medicine, Seoul 03722, Republic of Korea; dkdldslak@yuhs.ac; 2 Department of Physical and Rehabilitation Medicine, Center for Prevention and Rehabilitation, Heart Vascular Stroke Institute, Samsung Medical Center, School of Medicine, Sungkyunkwan University, Seoul 06351, Republic of Korea; hohoho7490@gmail.com; 3 School of Mechanical Engineering, Yonsei University, Seoul 03722, Republic of Korea; seeun.kim@sfa.co.kr (S.K.); jhchoi@doamtop.co.kr (J.H.C.); ysbaek@yonsei.ac.kr (Y.S.B.); 4 Construction Robot and Automation Laboratory, Department of Civil and Environmental Engineering, Yonsei University, Seoul 03722, Republic of Korea; junghoon@yonsei.ac.kr; 5 Department of Rehabilitation Medicine, Konyang University College of Medicine, Daejeon 35365, Republic of Korea; jbocean@kyuh.ac.kr; 6 Department of Rehabilitation Medicine, Gangnam Severance Hospital, Yonsei University College of Medicine, Seoul 06273, Republic of Korea; drtlc@yuhs.ac; 7 Department and Research Institute of Rehabilitation Medicine, Yonsei University College of Medicine, Seoul 03722, Republic of Korea

**Keywords:** stroke, step length asymmetry, gait asymmetry, weight shifting, gait training

## Abstract

Step length asymmetry is a characteristic feature of gait in post-stroke patients. A novel anterioposterior weight-shift training method with visual biofeedback (AP training) was developed to improve the forward progression of the trunk. This study aimed to investigate the effect of AP training on gait asymmetries, patterns, and gait-related function in subacute stroke patients. Forty-six subacute stroke patients were randomly assigned to the AP training group or the control group. The AP training group received conventional gait training and AP training five times per week for 4 weeks. The control group received the same intensity of conventional gait training with patient education for self-anterior weight shifting. Plantar pressure analysis, gait analysis, energy consumption, and gait-related behavioral parameters were assessed before and after training. The AP training group showed significant improvement in step length asymmetry, forefoot contact area and pressure, Berg balance scale score, and Fugl-Meyer assessment scale of lower extremity score compared to the control group (*p* < 0.05). However, there was no significant between-group difference with respect to energy cost and kinetic and kinematic gait parameters. In conclusion, AP training may help improve the asymmetric step length in stroke patients, and also improve anterior weight shifting, balance, and motor function in subacute stroke survivors.

## 1. Introduction

Post-stroke hemiparetic gait is characterized by an asymmetric pattern of walking [1,2,3]. Gait asymmetry can lead to comorbidities further affecting post-stroke gait such as slow walking speed [1], impaired balance [2], loss of bone density [3], musculoskeletal injuries, and joint pain [4]. Among these, correction of step length asymmetry is an important aspect of post-stroke gait rehabilitation after the patient has regained the ability to walk independently [1,5,6,7].

The shortened non-paretic step length is associated with impaired forward propulsion, which is generated through the anterior–posterior ground force of the paretic side that enables the trunk to progress forward while the non-paretic side is in swing [8]. The ground reaction force is the force generated by the weight-bearing surface, and post-stroke patients typically have weight-bearing asymmetry with a shift in the mean position of the center of pressure (CoP) toward the non-paretic side [9].

Recent studies have demonstrated that visual feedback can promote weight shifting in stroke patients, improving asymmetric posture [10], walking velocity [11], and balance control [11]. However, most of these studies entailed multi-directional weight-shift training, and subjects had chronic stroke or other disorders. Moreover, no randomized controlled trials (RCTs) have investigated the therapeutic effect of improvements in gait asymmetry on walking in patients with subacute stroke.

Our study group developed an anterioposterior weight-shift training system with visual feedback (AP training) to improve step length asymmetry [12]. This training system was designed based on the pathologic mechanism, i.e., impaired forward propulsion on the paretic side that enables the trunk to progress forward. Forward propulsion can be assessed by measuring real-time CoP trajectory using a plantar pressure analysis system, and this training system helps promote anterior weight shifting on the affected side using visual biofeedback. In our previous preliminary case series study, this training system was found to improve step length asymmetry in chronic stroke patients [12].

Because most of the functional gains tend to be achieved during the first 12 weeks after stroke [13], we hypothesized that AP training would be more effective in the subacute phase of stroke. Therefore, we conducted a single-blind RCT to investigate the effect of AP training on gait asymmetry in acute stroke patients primarily, and to investigate whether AP training is effective in improving plantar pressure distribution, gait pattern, and gait-related motor function.

## 2. Materials and Methods

### 2.1. Participants

Forty-six post-stroke patients admitted to the Department of Rehabilitation Medicine at Severance Hospital, Yonsei University College of Medicine, between June 2014 and June 2020 were enrolled.

The inclusion criteria were (1) hemiplegic patients who had a stroke within the preceding six months; (2) an ability to walk independently for 10 m; (3) Korean Mini Mental State Examination score ≥ 15; (4) asymmetrical gait pattern with a step length asymmetry ratio (SLAR) > 1.1; (5) provision of written informed consent for participation in the study; (6) ability to understand the purpose of this study and to adapt to the treatment process; and (7) age > 19 years. The exclusion criteria were (1) patients with quadriplegia or double hemiplegia; (2) a musculoskeletal or other nervous system disorder; (3) history of more than one stroke; or (4) patients judged to be unsuitable for participation for any other reason.

The sample size for this study was estimated as follows: A preliminary study involving five stroke patients was conducted to estimate the sample size required for the main study. The pre- and post-treatment SLARs and weight shifting were assessed. The change in the patients’ average SLAR in the preliminary study was 1.008 (±0.61). Therefore, the mean change in the SLAR value was assumed to be 1.008 in the treatment group and 0.4 in the control group (40% of that in the treatment group), and the standard deviation in both groups was assumed to be 0.61. Factoring a power of 80% and an alpha of 0.05, seventeen patients each were required for the treatment group and the control group. Assuming a dropout rate of 30%, a total of 46 patients were enrolled.

**Figure 1:**
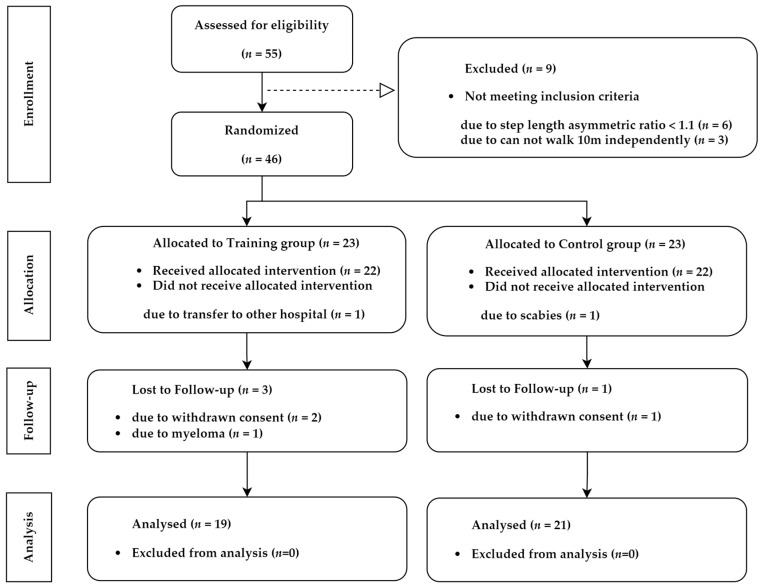
Participant consort flow diagram of study recruitment.

A schematic illustration of the study design and patient selection criteria is presented in Figure 1. Fifty-five patients were assessed for eligibility, of which nine patients were excluded because they did not qualify the inclusion criteria. A total of 46 patients were randomly assigned to the training group (*n* = 23) and control group (*n* = 23). In the training group, 1 patient dropped out during training, and 3 patients were lost to follow-up. In the control group, 1 patient dropped out during training and 1 patient was lost to follow-up. Finally, 19 patients in the training group and 21 patients in the control group were analyzed.

A total of 40 patients completed training and assessment (AP training group, *n* = 19; control group, *n* = 21) with no adverse events. At baseline, there were no significant between-group differences in terms of age, sex, lesion side of stroke, type of stroke, duration, Fugl-Meyer assessment (FMA), functional independent measure (FIM), Berg balance scale (BBS) score, maximum safe walking speed (MSWS), self-selected walking speed (SSWS), timed up and go (TUG), step length asymmetric index (SLAI), and SLAR (Table 1).

### 2.2. Study Design

**Figure 2:**
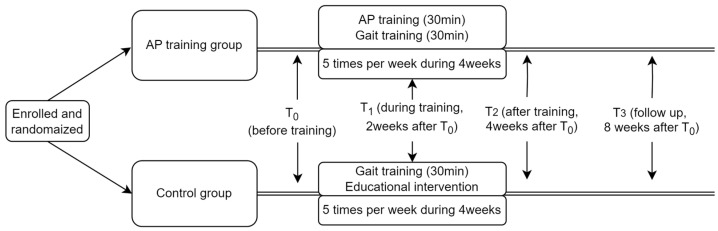
Study design.

This was a single-blind RCT. Participants were randomly assigned to the AP training group or the control group. Both groups received 30 m of conventional gait training for 4 weeks. The AP training group additionally received 30 m of AP training 5 times per week for 4 weeks. The control group received education regarding weight shifting to improve forward progression using the paretic lower limb and were encouraged to perform weight-shift training themselves at their bedside, but did not receive visual feedback training (Figure 2).

The step length parameters, behavior parameters, and plantar pressure analysis parameters of all patients were assessed before training (T_0_), during training (2 weeks after T_0_, T_1_), after training (4 weeks after T_0_, T_2_), and at follow-up (4 weeks after training, T_3_). Three-dimensional motion analysis and energy consumption were assessed at T0 and T2 (Table 2).

### 2.3. Anterioposterior Weight-Shift Training Using Visual Feedback

In this study, we used an AP training system developed by our study group to provide real-time feedback to patients about how they were shifting their weight anteriorly by measuring and processing foot pressure in real time using an F-Scan hardware system and software development kit (Tekscan, Inc., South Boston, MA, USA) [12]. Each training unit was divided into an evaluation session and a training session. Before starting the training unit, each participant attached a motion tracker to the ankle and pelvis of the affected side to measure the posture. The tracker was used to prevent the patients from using a compensatory posture of bending the knee when shifting weight to the affected side [12].

First, the participants underwent the evaluation session. In the basic position, the patient placed the affected foot 30 cm in front of the unaffected foot with both legs shoulder-width apart. In the evaluation session, subjects were asked to shift their weight anteriorly onto their affected foot as much as possible while receiving visual feedback regarding how much pressure was applied to each foot. The CoP trajectory of the feet was measured 10 times. A target value was set by adding 5% to the average of the CoP trajectories.

**Figure 3:**
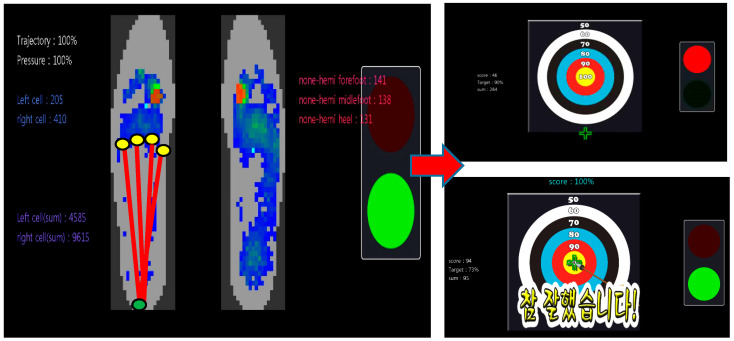
Anterioposterior weight-shift training using visual feedback—training session. The score on the archery target shows how much weight the patient is bearing on the affected foot, and also the extent to which the CoP trajectory has shifted anteriorly. Red line indicated the trajectory moving forward and green symbol was real-time CoP.

In the training session, subjects were asked to shift their weight anteriorly onto their affected foot as much as possible while receiving visual feedback regarding how much trajectory movement was applied to the affected foot and then move their unaffected foot forward one step. When the CoP trajectory reached the target value, an arrow was placed in the center of the target board, corresponding to 100 points, and the phrase “Good job” was displayed on the monitor and played over the system’s speakers (Figure 3). Then, the patients had to continue moving until they were in the correct position, which was when the pelvis had moved ahead of the ankle malleolus, as determined through the hip and ankle sensors. If the CoP trajectory had not reached the target value, an arrow appeared corresponding to the trajectory percentage, and the phrase “Try harder” was displayed on the monitor and played over the system’s speakers. If the participant’s pelvis was not far enough ahead, the phrase “Keep going to get the correct posture” was displayed on the monitor and played over the system’s speakers. Each training session lasted 30 m and consisted of two rounds, each consisting of 10 m of training and 5 m of rest.

### 2.4. Outcome Measurements

#### 2.4.1. Primary Outcomes

##### Gait Asymmetry

Step length parameters including SLAI, SLAR, affected side-step length (ASL), and unaffected side-step length (USL) were obtained using the HMER4 body pressure measurement system (Tekscan, Inc., Norwood, MA, USA) [1,14]. During each training session, 10 footstep images were obtained by making the participants walk 10 times across a 231.2 cm × 88.4 cm sensing area which had 7072 force-sensing resistors, corresponding to 0.3 sensors per cm^2^ [14]. The average of the images was used for analysis. The step length asymmetric index was calculated according to the following formula:

(paretic step length − non-paretic step length)/(0.5 × (paretic step length + non-paretic step length))

The asymmetry indices for stance time, swing time, double support time, and the intra-limb ratio of swing and stance were also calculated using temporospatial data obtained from a 3D motion analysis system (Vicon Peak, Englewood, CO, USA).

#### 2.4.2. Secondary Outcomes

##### Plantar Pressure Analysis

Contact area, contact pressure, trajectory length, and the number of back movements (NOB) were measured using insole pressure as measured by an F-Scan plantar pressure measurement system (Tekscan, Inc., Norwood, MA, USA). Insoles were trimmed to each participant’s foot size and placed in each of their shoes after removing the original insoles. Data were measured after making the participants walk for 30 m. Contact area was measured on the forefoot, midfoot, hindfoot, and total. Contact pressure was measured on the forefoot, midfoot, hindfoot, total, and peak pressure. Trajectory was measured in the anterioposterior and mediolateral directions.

##### Behavioral Parameters

The behavioral parameters assessed were functional ambulatory category (FAC), FMA scale score [15], functional independent measure (FIM), medical record council (MRC) grading of muscle power for lower extremities, SSWS, MSWS [16], TUG, and BBS score [17]. All participants were evaluated by an occupational therapist. SSWS was calculated by measuring the time required by participants to walk 10 m on flat ground at their usual speed; the average value from three measurements was calculated to obtain the SSWS. MSWS was calculated in the same way as SSWS except the participants were asked to walk as quickly as possible. TUG was measured by asking participants to begin in a sitting position, stand up, walk 3 m, turn around, and then sit again. This was repeated 3 times and the average duration was used in the analysis [17].

##### Energy Consumption and 3D Motion Analysis

Energy consumption was assessed using the following parameters: O_2_ cost (mL/kg/m) and O_2_ rate (mL/min/kg). Energy consumption was measured every 30 s while the participants walked around a 20 m oval track with bare feet at a comfortable speed for 5 min using a KB1-C system (Aerosports, Inc., Ann Arbor, MI, USA). The O_2_ rate was obtained using the average value collected over 3–5 min [18].

The 3D motion analysis included the temporospatial parameters, kinematic parameters, and kinetic parameters. These parameters were obtained by recording participants walking 8 m at the selected speed 3 times using the VICON MX-T10 Motion Analysis System (Oxford Parameters, Inc., Oxford, UK) [12]. The average value of the five walking cycles was used for analysis.

### 2.5. Statistical Analysis

The characteristic analysis used two-sample *t*-test or descriptive statistics. All variables were analyzed using the repeated-measure analysis of variance (RMANOVA) with post hoc test with Bonferroni correction. *p* < 0.05 was interpreted as a meaningful result. SPSS version 20.0 for Windows (SPSS, Chicago, IL, USA) was used for all statistical analyses.

## 3. Results

### 3.1. Primary Outcomes

**Figure 4:**
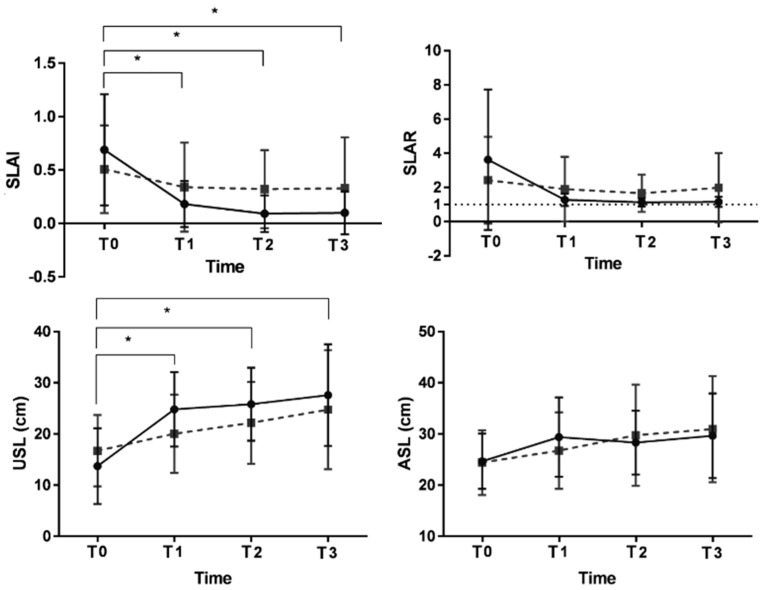
Comparison of step length parameters measured by BPMS through a time × intervention factor interaction post hoc test between the training group (solid line) and control group (dotted line) before training (T0), during training (T1), after training (T2), and at post-training 4-week follow up (T3). * *p* < 0.0125 is statistically significant for time × intervention interaction according to post hoc tests. SLAI, step length asymmetric index; SLAR, step length asymmetric ratio; USL, unaffected step length; ASL, affected step length.

At baseline, there were no significant differences between the AP training group and the control group with respect to asymmetrical gait parameters. Repeated-measures analysis of variance revealed a significant interaction between timeand intervention with regard to SLAI (F(6.160), *p* = 0.008) and USL (F(4.929), *p* = 0.009), indicating that the AP training group showed a greater increase in SLAI and USL over time than the control group (Figure 4). Post hoc comparisons showed that in the AP training group, SLAI was better at T1, T2, and T3 than at T0 (*p* = 0.000, 0.000, and 0.000, respectively), indicating that the SLAI increased during the treatment period and this effect was maintained for 4 weeks after training. However, the asymmetry indices of stance time, swing time, double support time, and swing/stance time were not significantly different between the groups over time (Table 3).

### 3.2. Secondary Outcomes

#### 3.2.1. Plantar Pressure Analysis

The RMANOVA test of forefoot contact area, midfoot contact area, and total foot contact area on the affected side revealed a significant interaction between time and intervention (F(3.118), *p* = 0.040; F 4.673, *p* = 0.009; and F 3.832, *p* = 0.021, respectively), indicating a significant improvement in the forefoot, midfoot, and total foot contact area after training in the AP training group compared to the control group (Table 4). The RMANOVA test of forefoot contact pressure, midfoot contact pressure, and total foot contact pressure on the affected side also revealed a significant interaction between time and intervention (F(4.307), *p* = 0.014; F(4.394), *p* = 0.010; and F(4.307), *p* = 0.015, respectively). This indicated a significant improvement in forefoot pressure, midfoot pressure, and total foot contact pressure after training in the AP training group compared to the control group. Post hoc comparisons showed that, compared to T0, at T1, T2, and T3, the forefoot contact area (*p* = 0.031, 0.001, 0.004, respectively), midfoot contact area (*p* = 0.011, 0.001, 0.003, respectively), total foot contact area (*p* = 0.003, 0.001, 0.004, respectively), forefoot contact pressure (*p* = 0.001, 0.000, 0.000, respectively), midfoot contact pressure (*p* = 0.042, 0.003, 0.001, respectively), and total foot contact pressure (*p* = 0.002, 0.014, 0.009, respectively) were significantly increased after training in the AP training group than the control group. These results indicated that the AP training group had significantly better forefoot, midfoot, and total contact area and contact pressure during training and that this improvement was maintained at 4 weeks after the completion of training. However, hindfoot contact area, hindfoot contact pressure, and peak contact pressure were not significantly different between the two groups over time.

On the unaffected side, the RMAONOVA tests revealed a significant interaction between time and intervention for forefoot contact area (F(2.937), *p* = 0.036) (Table 4). Post hoc comparisons showed that, compared to T0, the AP training group had significantly higher forefoot contact area at T2, T3, and T4 (*p* = 0.017, 0.045, 0.156, respectively). The other parameters did not differ significantly between the groups over time.

With regard to foot scan trajectory, RMANOVA revealed a significant interaction between time and intervention) for the affected side AP trajectory (F(5.372), *p* = 0.003), indicating that the affected side AP trajectory was significantly increased in the AP training group than the control group and showed a tendency to increase on the unaffected side. Post hoc comparisons showed that, compared to T0, at T1, T2, and T3, the affected side AP trajectory (*p* = 0.002, 0.001, 0.001, respectively) increased more in the AP training group than in the control group. This indicated that the AP training group showed a significant improvement in the AP trajectory during training and that this improvement persisted for 4 weeks after the completion of training. The other foot scan trajectory parameters were not significantly different between the groups.

#### 3.2.2. Behavioral Parameters

At T0, there was no significant difference between the two groups regarding the clinical evaluation parameters (Table 5). RMANOVAs revealed a significant interaction between time and intervention for knee extensor MRC scale score, ankle dorsiflexor MRC score, FMA, and BBS (F(4.626), *p* = 0.007; F(3.579), *p* = 0.036; F(3.276), *p* = 0.033; F(5.738), *p* = 0.005, respectively). This indicated that the AP training group showed a significant improvement in knee extensor MRC scores, ankle dorsiflexor MRC scores, FMA, and BBS during training than the control group and that this improvement was maintained for 4 weeks after the completion of training. Post hoc comparisons showed that, compared with T0, at T1, T2, and T3, the knee extensor MRC score (*p* = 0.031, 0.001, 0.004, respectively), ankle dorsiflexor MRC score (*p* = 0.011, 0.001, 0.003, respectively), FMA (*p* = 0.003, 0.001, 0.004, respectively), and BBS (*p* = 0.001, 0.000, 0.000, respectively) were better in the AP training group than the control group. This indicated that the AP training group had significantly better knee extensor MRC scale scores, ankle dorsiflexor MRC scores, FMAs, and BBSs during AP training, and that this improvement was maintained at 4 weeks after the completion of training. However, MSWS, SSWS, FAC, and FIM were not significantly different between the groups over time.

#### 3.2.3. Energy Consumption and 3D Motion Analysis

At T0, there was no significant between-group difference with respect to energy consumption (Table 6). RMANOVAs revealed a significant interaction between time _(T0, T3)_ and intervention for O_2_ cost (F(3.213), *p* = 0.042), indicating that the control group had a significantly decreased O_2_ cost compared to the AP training group.

In terms of temporospatial, kinetic, and kinematic parameters, there were no significant differences between the two groups at baseline or over time (Supplementary Tables S1–S3).

## 4. Discussion

To the best of our knowledge, this is the first RCT evaluating biofeedback in anterioposterior weight-shifting training in patients with subacute stroke. The recovery aspects in stroke patients vary depending on the phase (acute or chronic) [19]. Importantly, early gait recovery after stroke is associated with future gait independence [20,21]. In this study, subacute stroke patients who received AP training with traditional rehabilitation services showed significant improvements in step length asymmetry, forefoot contact area and pressure, Berg balance scale score, and Fugl-Meyer assessment scale of lower extremity compared to their counterparts who received only educational intervention with traditional rehabilitation.

This study demonstrated the effectiveness of AP training for improving asymmetric step length. This was consistent with previous studies in which repetitive weight-shifting training on the affected side was effective in improving step length asymmetry using compelled weight-shift training [22] and body weight support training [23]. However, in the study by Sheikh et al., gait training combined with compelled body-weight-shift therapy via a shoe lift applied under the non-paretic leg was not significantly better than gait training alone in improving gait symmetry [24]. It was suggested that the training would have been more effective by increasing the use of paretic limbs in functional activities than intervention on the unaffected side [24].

Interactive rehabilitation programs potentially entail an effective adaptive motor learning process resulting in better functional outcomes [25,26], and visual feedback is one form of effective interactive training [27]. Reismane et al. emphasized the importance of motor adaptation through repetitive training to improve gait asymmetry [28]. In the present study, AP training included the appropriate use of “functional activity”, “biofeedback”, and “repetitive gait training”, which were emphasized in previous studies. AP training induces the patient to repeat the gait cycle (stance phase on the affected side) with a forward weight shift to the affected side while playing an archery game with biofeedback. All these factors explain the better step length symmetry in the AP training group compared to the control group that received only educational intervention, and this effect was maintained until 4 weeks after training. A diverse range of factors can affect asymmetrical step length, and many studies have suggested that it is important to improve step length asymmetry along with proper weight transfer while walking [9,29,30]. In the present study, the AP training group showed significantly improved contact area and contact pressure for the anterior two-thirds of the feet and a greater affected side AP trajectory than the control group. These findings support the idea that AP training increases the propulsive force during the stance phase.

We also found an improvement in step length asymmetry with proper weight distribution in the AP training group. This was consistent with previous studies in which weight-shift training with biofeedback enabled more effective weight distribution [31,32]. In the study by Nunzio et al., participants who received weight-shift training with biofeedback had a significantly better CoP asymmetry index than those who did not [31]. In contrast to our findings, a meta-analysis of visual feedback training in standing position in acute and subacute stroke subjects showed no significant effect on weight distribution, postural sway, or gait compared to conventional therapy [33]. This is in line with Sunkarat’s suggestions that visual feedback training in standing position may not translate to improved performance during gait and gait-related activities [34]. However, recent studies have demonstrated the effect of visual feedback training in improving weight distribution [32,35]. Pak et al. found that visual feedback training of stroke patients through the use of visual targets was significantly effective in improving the weight-bearing proportion of the affected side compared with the control group [35]. With regard to gait disorders, visual information can be used to compensate for inappropriate proprioception and help correct body asymmetry through the reorganization of visuomotor information [32].

Van Peppen et al. indicated that training in postural control should be applied while performing gait-related tasks [33]. This is similar to the importance of motor adaptation in functional activities, and AP training was found to be an appropriate training method for performing gait-related tasks [28,36,37].

With respect to the behavior parameters, the AP training group in this study showed significantly better balance control with improved lower extremity motor ability than the control group. This was similar to the findings of previous studies in which visual feedback training was found to improve balance and motor function [12,38,39,40]. Lauziere et al. reported a strong relationship between motor function of the paretic lower extremity, balance control, weight bearing distribution, and gait asymmetry in stroke patients [5]. Hsu et al. also reported that ankle dorsiflexor strength, ankle plantarflexor strength, knee extensor strength, plantarflexor peak torque, and motor function of the paretic lower extremity measured with FMA significantly correlated with spatiotemporal asymmetry [41]. Lower extremity function is important for the relationship of walking efficiency with symmetrical gait characteristics in stroke patients [38]. Lewek et al. examined the relationship between spatiotemporal gait asymmetry and balance in post-stroke patients. They found a correlation between step length asymmetries and BBS scores, suggesting that gait asymmetries are associated with the risk of falls in these individuals [2]. Asymmetric weight bearing and increased compensation from the non-paretic leg contribute to the deficits in balance control commonly observed after stroke [19]. In a study, visual feedback rhythmic weight-shift training (balance master) was found to improve dynamic balance function in hemiplegic stroke patients, resulting in a lower incidence of falls, although not statistically significant [39]. The improvements in weight distribution, symmetric gait pattern, and lower extremity motor ability in stroke patients who received the AP training may have influenced the improvement in balance ability.

Contrary to our expectations, we did not observe any improvement in walking speed, energy consumption, temporal asymmetry, or kinetic and kinematic parameters. With respect to walking speed, the AP training group tended to show an improved average maximum walking speed by 0.1 m/s compared to the control group, but the between-group difference was not statistically significant. Previous descriptive cross-sectional studies have reported the relationship between step length asymmetry and walking speed [1,40]. However, the effect of training on walking speed is still unclear. In the study by Pak et al., the walking speed in the visual feedback training group was not significantly increased compared to the control group [35]. Sheikh et al. also found that gait training combined with compelled body-weight-shift training was not significantly better than gait training alone in terms of improving velocity in patients with chronic stroke [24]. Robotic-assisted body-weight-supported treadmill training with visual feedback also improved gait symmetry but did not significantly improve gait velocity and endurance [42]. However, in the studies by Sungkarat et al. and Aruin et al., a lift insert in conjunction with physical therapy significantly increased the gait velocity compared with the control group [34]. In summary, most RCTs using weight-shift training with visual feedback found no significant improvement in gait velocity, except for some studies using insoles.

With respect to energy consumption, improving step length symmetry with visual feedback had no significant effect on metabolic cost, which is consistent with previous studies [6,30]. Moreover, a previous study found no definitive evidence about the relationship between energy expenditure and spatiotemporal symmetry in stroke patients [5]. Padmanabhan et al. demonstrated that post-stroke patients often retain the ability to walk with symmetric step lengths (symmetric steps); however, the resulting walking pattern remains effortful [6]. Wutzke et al. argued that long-term correction and learning are necessary for symmetrical walking with reduced energy consumption [29]. Indeed, stroke patients who received the AP training tended to walk more carefully to achieve gait symmetry than the control group. We believe that the duration of training in this study may be relatively short to achieve full motor learning. To reduce energy consumption and increase walking speed, a longer training protocol will be needed to reach automatization without attention.

We expected that improved asymmetric step length leads to changes in kinematic parameters in the sagittal plane, as a previous study suggested an important role of the plantar flexor muscles and hip flexor muscles in step length asymmetry [5]. However, we could not find any significant difference in the kinematic parameters between groups in this study. Despite there being no comparative studies with visual feedback training, this study is in line with some previous studies with robot-assisted gait training for improving step length that did not confirm the significance of kinematic parameters between groups [43,44]. It may be in line with previous suggestions that a simple performance of a pattern does not appear to be sufficient to change the pattern over the longer term. Studies in both animals and humans indicate that changes in, for example, corticospinal excitability or motor maps occur only with practice of skilled movement and not with mere use [45,46]. In another interpretation, Nikamp et al. explained that 3D gait analysis measured in a specialized gait laboratory was affected by walking parameters [47], and it is also necessary to consider whether the unfamiliar environment affected the patient’s gait during the 3D gait analysis.

Some limitations of this study should be considered while interpreting the results. First, all participants were receiving acute management in the hospital. Therefore, they received a lot of interventions during the study period despite our effort to provide the same intervention intensity for all study participants to minimize bias. Second, we did not confirm an improvement in temporal asymmetry. A future study with a larger sample size may be required to assess this aspect because other behavioral parameters related to temporal asymmetry were significantly improved in the AP training group. Third, the training period in this study was only 4 weeks. The study period was in consideration of the period of hospitalization for acute rehabilitation in Korea; however, if possible, a long-term training study will be needed to confirm the effects of gait pattern, energy consumption, etc.

This study investigated a training method tailored to the latest research trends. The comprehensive analysis of various data, such as 3D motion analysis, plantar pressure analysis, and clinical data, was a strength of the study. Not many studies have performed such comprehensive analyses. Our findings may help design future RCTs.

Our research focus was on visual feedback, but recent studies have also demonstrated the effectiveness of tactile and detailed auditory feedback in helping improve the gait patterns of stroke patients [48,49]. Ma et al. reported an immediate effect of a wearable vibro-tactile biofeedback device on plantar loading and gait pattern in chronic stroke patients [50]. Therefore, further research should determine how best to combine visual, tactile, and auditory biofeedback to maximize the effectiveness of stroke gait rehabilitation. Recently, many wireless trackers and pressure sensors have been developed. Further research using wireless equipment may facilitate the gait rehabilitation of stroke patients.

## 5. Conclusions

In this RCT, AP training was found to significantly improve step length asymmetry, weight disturbance, balance control, and motor function of the lower extremity compared to the control group, and the improvement was maintained for 4 weeks after training. However, there were no significant between-group differences in terms of temporal asymmetry, energy consumption, or kinetic and kinematic parameters. Clinicians may consider weight-shift training with biofeedback in all patients with shorter non-paretic step lengths during gait rehabilitation.

## Figures and Tables

**Table 1:** Baseline characteristics of subjects.

Characteristic	AP Training Group (*n* = 19)	Control Group (*n* = 21)	*p* Value
Age (years)	57.7 ± 17.7	52.0 ± 14.0	0.267
Sex (male)	10 (52.6)	14 (66.7)	0.366
Lesion side of stroke (left)	9 (47.4)	11 (52.4)	0.752
Type of stroke			0.141
Ischemic	15 (78.9)	12 (57.1)	
Hemorrhagic	4 (21.1)	9 (42.9)	
FAC			0.587
3	13 (68.4)	14 (66.7)	
4	6 (31.5)	7 (33.3)	
Duration from onset	97.0 ± 59.8	81.1 ± 54.2	0.383
FMA	39.9 ± 17.0	43.6 ± 22.1	0.558
FIM_mobility	18.4 ± 2.7	17.1 ± 4.1	0.255
BBS	25.4 ± 7.3	24.6 ± 7.9	0.756
MSWS (m/s)	0.3 ± 0.1	0.2 ± 0.1	0.408
SSWS (m/s)	0.2 ± 0.1	0.2± 0.1	0.869
TUG (s)	46.7 ± 24.6	56.1 ± 64.1	0.555
SLAR	3.6 ± 4.1	2.5 ± 2.5	0.301
SLAI	0.7 ± 0.5	0.5 ± 0.4	0.285

Data presented as mean ± standard deviation or frequency (%); FAC, functional ambulation category; FMA, Fugl-Meyer assessment; FIM, functional independent measure; BBS, Berg balance scale; MSWS, maximum safe walking speed; SSWS, self-selected walking speed; TUG, timed up and go; SLAI, step length asymmetric index; SLAR, step length asymmetric ratio.

**Table 2:** Timepoints for measurement of parameters.

	Before Training	During Training	After Training	Follow Up
Primary outcome				
Gait asymmetry	V	V	V	V
Secondary outcomes				
Plantar pressure analysis	V	V	V	V
Behavior parameters	V	V	V	V
3D motion analysis	V		V	
Energy consumption	V		V	

“V” represents the measuring timepoints.

**Table 3:** Comparison of asymmetric indices of temporospatial walking parameters.

	AP Training Group		Control Group		
Asymmetric Index	Before Training	After Training	Before Training	After Training	*p* Value
Stance time (s)	−0.10 ± 0.08	−0.14 ± 0.11	−0.15 ± 0.09	−0.16 ± 0.09	0.666
Swing time (s)	0.60 ± 0.34	0.50 ± 0.29	0.61 ± 0.30	0.49 ± 0.24	0.679
Double support time (s)	0.01 ± 0.04	0.01 ± 0.06	0.01 ± 0.06	0.00 ± 0.07	0.666
SW/ST	0.69 ± 0.37	0.61 ± 0.32	0.74 ± 0.30	0.63 ± 0.26	0.634

Data presented as mean ± standard deviation; SW/ST, swing/stance time.

**Table 4:** Comparison of plantar pressure analysis.

	AP Training Group				Control Group				*p* Value
	Before Training	During Training	After Training	Follow Up	Before Training	During Training	After Training	Follow Up	
Affected side									
Contact area (mm^2^)									
Forefoot	27.1 ± 30.6	39.2 ± 24.5	57.5 ± 34.9 *	56.0 ± 42.1 *	26.3 ± 21.0	20.7 ± 21.8	32.7 ± 26.5	40.8 ± 28.7	0.029
Midfoot	92.7 ± 49.0	120.6 ± 4 *	149.7 ± 54.2 *	150.7 ± 66.0 *	106.4 ± 64.0	99.2 ± 53.0	116.0 ± 52.5	117.0 ± 58.0	0.004
Hindfoot	119.6 ± 40.5	131.0 ± 50.7	125.2 ± 51.4	129.3 ± 43.4	103.0 ± 33.1	97.1 ± 38.1	108.8 ± 34.1	97.4 ± 40.5	0.189
Total	239.5 ± 93.5	290.8 ± 89.6 *	331.9 ± 111.9 *	337.4 ± 132.4 *	235.5 ± 90.1	216.9 ± 89.4	257.5 ± 82.5	255.2 ± 91.1	0.012
Contact pressure (kPa)									
Forefoot	26.4 ± 34.6	51.7 ± 46.9 *	80.0 ± 64.6 *	80.7 ± 63.3 *	25.3 ± 18.05	22.0 ± 19.1	32.9 ± 25.0	48.4 ± 47.3	0.006
Midfoot	96.4 ± 54.9	140.8 ± 73.4	173.4 ± 76.4 *	202.9 ± 107.3 *	135.9 ± 96.7	143.5 ± 84.3	159.4 ± 89.8	152.7 ± 94.6	0.006
Hindfoot	185.2 ± 80.2	213.2 ± 88.7	190.5 ± 67.0	211.6 ± 114.2	181.1 ± 97.6	174.5 ± 101.8	180.2 ± 88.6	173.4 ± 117.7	0.523
Total	308.1 ± 92.7	400.4 ± 84.8 *	420.0 ± 121.3	487.4 ± 214.0 *	342.4 ± 82.3	339.5 ± 86.3	372.1 ± 90.1	374.3 ± 159.9	0.033
Peak	418.7 ± 134.4	477.8 ± 118.6	489.5 ± 161.8	566.1 ± 256.3	430.7 ± 99.0	417.5 ± 94.7	473.7 ± 152.2	479.6 ± 163.4	0.306
Trajectory (mm)									
AP	43.3 ± 65.9	72.6 ± 56.0 *	78.6 ± 56.9 *	91.1 ± 57.3 *	70.6 ± 50.1	64.4 ± 51.5	68.8 ± 63.1	69.0 ± 61.1	0.001
ML	6.3 ± 5.0	9.2 ± 7.8	10.2 ± 8.0 *	10.8 ± 8.1 *	10.9 ± 6.5	9.7 ± 7.3	8.3 ± 4.9	8.7 ± 7.1	0.018
NOB	1.4 ± 0.6	1.6 ± 0.8	1.5 ± 0.7	1.6 ± 0.9	1.3 ± 0.7	1.7 ± 0.7	1.3 ± 0.5	1.3 ± 0.5	0.553
Unaffected side									
Contact area (mm^2^)									
Forefoot	69.7 ± 36.9	89.9 ± 43.3	84.9 ± 35.3	84.0 ± 34.31	82.4 ± 46.0	76.7 ± 38.3	73.9 ± 38.1	78.5 ± 32.1	0.036
Midfoot	146.4 ± 56.3	164.9 ± 55.2	162.1 ± 57.7	158.7 ± 68.4	177.0 ± 49.9	174.0 ± 51.3	161.0 ± 50.0	168.3 ± 55.0	0.177
Hindfoot	126.5 ± 31.1	138.5 ± 33.7	135.1 ± 18.6	132.7 ± 29.2	139.5 ± 26.7	139.2 ± 26.7	137.0 ± 20.8	127.1 ± 29.2	0.269
Total	342.4 ± 106.5	393.3 ± 119.9	382.0 ± 88.4	375.3 ± 115.5	398.9 ± 104.0	389.9 ± 99.7	372.0 ± 83.1	374.1 ± 103.1	0.071
Contact pressure (kPa)									
Forefoot	113.5 ± 70.9	124.8 ± 56.8	155.4 ± 97.6	148.3 ± 86.7	120.6 ± 73.7	104.6 ± 66.8	122.7 ± 91.2	127.0 ± 64.2	0.267
Midfoot	180.0 ± 93.2	194.2 ± 93.4	225.1 ± 103.1	217.1 ± 95.7	194.6 ± 58.3	205.3 ± 101.7	200.4 ± 94.3	207.6 ± 84.3	0.411
Hindfoot	239.2 ± 105.3	229.9 ± 63.5	237.0 ± 49.9	254.7 ± 146.7	247.4 ± 69.4	231.3 ± 78.6	272.2 ± 72.7	223.4 ± 95.4	0.272
Total	532.8 ± 213.3	549.0 ± 178.2	608.7 ± 176.8	620.2 ± 222.7	562.6 ± 143.5	540.9 ± 185.7	595.3 ± 174.0	558.2 ± 179.4	0.528
Peak	701.4 ± 275.8	667.5 ± 228.5	758.7 ± 270.1	753.2 ± 268.2	716.0 ± 210.9	678.5 ± 243.2	734.9 ± 216.8	710.4 ± 233.9	0.866
Trajectory (mm)									
AP	140.0 ± 29.9	145.2 ± 42.7	155.0 ± 26.4	154.5 ± 23.1	159.7 ± 24.0	157.5 ± 26.8	141.4 ± 39.3	153.0 ± 33.2	0.053
ML	7.3 ± 5.8	7.3 ± 5.4	7.8 ± 6.0	6.7 ± 5.7	8.4 ± 6.7	6.6 ± 4.3	9.9 ± 7.7	9.1 ± 4.6	0.621
NOB	2.6 ± 2.6	2.4 ± 1.6	2.5 ± 1.5	2.4 ± 1.7	2.5 ± 1.3	2.5 ± 1.1	2.1 ± 1.0	2.4 ± 1.2	0.739

Data presented as mean ± standard deviation; * Adjusted *p*-values < 0.05 are statistically significant for time × intervention interaction according to post hoc tests. AP, anterioposterior; ML, mediolateral; NOB, number of back.

**Table 5:** Comparison of gait-related behavioral parameters.

	AP Training Group				Control Group				*p* Value
	Before Training	During Training	After Training	Follow Up	Before Training	During Training	After Training	Follow Up	
MRC on									
Hip flexor	3.1 ± 0.6	3.2 ± 0.6	3.5 ± 0.8	3.7 ± 0.7	3.1 ± 0.7	3.3 ± 0.7	3.3 ± 0.7	3.5 ± 0.8	0.288
Hip extensor	3.3 ± 0.7	3.5 ± 0.8	3.7 ± 0.7	3.9 ± 0.7	3.1 ± 0.8	3.3 ± 0.8	3.3 ± 0.8	3.5 ± 0.8	0.340
Knee flexor	3.0 ± 0.7	3.2 ± 0.7	3.5 ± 0.8	3.7 ± 0.7	2.9 ± 0.8	3.1 ± 0.8	3.1 ± 0.9	3.3 ± 0.9	0.081
Knee extensor	3.2 ± 0.9	3.4 ± 0.8	3.7 ± 0.7 *	3.8 ± 0.8 *	3.2 ± 0.9	3.3 ± 0.7	3.2 ± 0.8	3.3 ± 1.0	0.007
Ankle dorsiflexor	1.8 ± 1.1	2.4 ± 1.1 *	2.6 ± 1.1 *	3.0 ± 1.4 *	2.0 ± 1.2	2.0 ± 1.2	2.2 ± 1.1	2.5 ± 1.2	0.036
Ankle plantarflexor	2.4 ± 1.2	2.8 ± 1.3	2.9 ± 1.3	3.2 ± 1.3	2.1 ± 1.4	2.2 ± 1.3	2.3 ± 1.3	2.6 ± 1.2	0.270
MSWS (m/s)	0.3 ± 0.1	0.4 ± 0.2	0.4 ± 0.3	0.5 ± 0.3	0.2 ± 0.1	0.3 ± 0.2	0.4 ± 0.2	0.4 ± 0.2	0.944
SSWS (m/s)	0.2 ± 0.1	0.3 ± 0.1	0.3 ± 0.2	0.4 ± 0.2	0.2 ± 0.1	0.3 ± 0.2	0.3 ± 0.2	0.4 ± 0.2	0.914
TUG (s)	46.8 ± 24.6	38.9 ± 24.2	34.3 ± 19.9	28.7 ± 10.7	56.1 ± 64.2	51.8 ± 55.6	43.1 ± 48.2	38.6 ± 31.1	0.836
FMA	39.9 ± 17.0	49.5 ± 20.9 *	54.7 ± 22.1 *	58.5 ± 20.6 *	43.7 ± 22.1	48.2 ± 22.3	51.4 ± 21.1	53.7 ± 21.9	0.033
FAC	3.3 ± 0.5	3.8 ± 0.6	4.2 ± 0.6	4.4 ± 0.6	3.3 ± 0.5	3.6 ± 0.6	3.9 ± 0.7	4.0 ± 0.7	0.210
FIM mobility	18.4 ± 2.7	21.3 ± 3.8	24.6 ± 4.6	26.6 ± 5.6	17.1 ± 4.1	19.1 ± 4.2	22.3 ± 5.5	24.4 ± 6.2	0.857
BBS	25.4 ± 7.3	36.8 ± 6.6 *	43.8 ± 6.9 *	45.0 ± 8.6 *	24.7 ± 7.9	29.3 ± 8.9	34.5 ± 9.6	38.3 ± 9.7	0.005

Data presented as mean ± standard deviation; * Adjusted *p*-values < 0.05 are statistically significant for time × intervention interaction according to post hoc tests. MRC, Medical Research Council; MSWS, maximum safe walking speed; SSWS, self-selected walking speed; TUG, timed up and go; FMA, Fugl-Meyer assessment; FAC, functional ambulation category; FIM, functional independent measure; BBS, Berg balance scale.

**Table 6:** Comparison of energy consumption.

Asymmetric Index	AP Training Group		Control Group		*p* Value
	Before Training	After Training	Before Training	After Training	
O_2_ cost (mL/kg/m)	0.7 ± 0.4	0.6 ± 0.4	0.8 ± 0.4	0.5 ± 0.3 *	0.042
O_2_ rate (mL/min/kg)	8.0 ± 2.3	7.9 ± 1.8	7.9 ± 1.7	7.8 ± 2.1	1.000

Data presented as mean ± standard deviation; * *p* < 0.05 is statistically significant for time × intervention interaction.

## Data Availability

The data presented in this study are available on request from the corresponding author. The data are not publicly available due to data protections regulations.

## Supplementary Materials

The following supporting information can be downloaded at https://www.mdpi.com/article/10.3390/jpm13121726/s1: Supplementary Table S1. Comparison of Temporospatial Walking Parameters; Supplementary Table S2. Comparison of Kinematic Parameters; Supplementary Table S3. Comparison of Kinetic Parameters.

## Abbreviations

The following abbreviations are used in this manuscript.

AP training	Anterioposterior weight-shift training with visual biofeedback
ASL	Affected side-step length
BBS	Berg balance scale
CoP	Center of pressure
FAC	Functional Ambulation Category
FIM-mobility	Functional Independent Measure-mobility
FMA	Fugl-Meyer assessment
K-MMSE	Korean Mini Mental State Examination
ML	Mediolateral
MRC	Medical Research Council scale
MSWS	Maximum safe walking speed
NOB	Number of back movement
RMANOVA	Repeated-measure analysis of variance
SLAI	Step length asymmetric index
SLAR	Step length asymmetric ratio
SSWS	Self-selected walking speed
TUG	Timed up and go test
USL	Unaffected side-step length
