# Supplementary material for: Effect of Anterioposterior Weight-Shift Training with Visual Biofeedback in Patients with Step Length Asymmetry after Subacute Stroke
Source: J Pers Med. 2023 Dec 18;13(12):1726. doi: 10.3390/jpm13121726 (PMC10745098; doi:10.3390/jpm13121726)
Supplement: Supplementary file 1 [file jpm-13-01726-s001.zip › Supplementary Tables.pdf]

Supplementary Table S1. Comparison of Temporospatial Walking Parameters

	AP training group		Control group		
	**Before training**	**After training**	**Before training**	**After training**	**P value**
Cadence (step/min)	57.4±14.1	62.6±20.3	53.2±15.6	58.8±19.1	0.809
Walking speed (m/s)	0.2±0.1	0.3±0.2	0.2±0.1	0.3±0.1	0.742
Stride time(s)	2.3±0.8	2.2±0.9	2.5±0.8	2.3±0.7	0.214
Step time (s)	1.2±0.3	1.2±0.5	1.3±0.4	1.2±0.5	0.271
Single support time (s)	0.3±0.1	0.3±0.1	0.3±0.1	0.3±0.1	0.876
Double support time (s)	1.5±0.8	1.3±0.9	1.6±0.8	1.3±0.7	0.574
Stance time (s)	1.7±0.8	1.6±0.9	1.8±0.9	1.5±0.7	0.370
Swing time (s)	0.6±0.3	0.6±0.2	0.7±0.4	0.7±0.5	0.316
Stride length (m)	0.4±0.1	0.5±0.2	0.4±0.1	0.5±0.2	0.298
Step length (m)	0.3±0.1	0.3±0.1	0.3±0.1	0.3±0.1	0.376

Data presented as mean ± standard deviation; * $P < 0.05$ was statistically significant for time × intervention interaction.

Supplementary Table S2. Comparison of Kinematic Parameters

	AP training group		Control group		
	**Before training**	**After training**	**Before training**	**After training**	**P value**
**Affected side**					
Pelvic max anterior tilit angle at ST	18.4±5.3	18.1±4.3	20.7±4.9	19.1±3.5	0.439
Hip max flexion angle at IC	27.6±11.7	28.7±9.0	28.8±8.7	27.9±5.7	0.400
Hip max flexion angle at SW	33.2±11.3	34.1±8.5	36.8±8.0	35.5±7.0	0.373
Hip min flexion angle at ST	13.1±10.6	10.1±10	13.1±8.6	10.4±7.9	0.925
Hip max abduction angle	3.1±5.6	3.6±4.6	5.3±4.2	5.3±3.4	0.778
Hip max adduction angle	-6.4±6.4	-6.6±3.8	-4.9±2.7	-4.7±3.0	0.750
Knee flexion angle at IC	15.6±8.1	13.8±7.3	14.1±7.7	12.3±6.3	0.997
Knee min flexion angle at ST	8.8±10.2	7.2±10.2	5.9±9.0	5.6±8.0	0.658
Knee max flexion angle at SW	34.2±13.2	38.6±13.5	33.6±11.2	33.0±10.0	0.142
Ankle DF angle at IC	-13.9±5.8	-13.8±6.1	-14.8±9.1	-13.2±8	0.362
Ankle max DF angle at ST	2.7±8.6	3.6±6.9	2.5±7.4	4.2±6.2	0.678
Ankle max PF at SW	-17.2±8.2	-16.8±7.3	-18.0±10.1	-14.4±8.4	0.095
**Unaffected side**					
Pelvic max anterior tilit angle at ST	18.8±5.4	18.2±4.2	21.2±5.4	19.3±3.8	0.442
Hip max flexion angle at IC	38.5±8.7	40.2±6.4	40.9±8.3	39.2±7.4	0.183
Hip max flexion angle at SW	39.8±9.4	42.6±6.3	43.0±9.3	42.8±6.5	0.185
Hip min flexion angle at ST	9.7±9.2	7.0±7.2	8.3±8.6	5.8±7.5	0.970
Hip max abduction angle	3.8±4.6	4.1±4.6	4.5±3.9	3.7±4.9	0.441
Hip max adduction angle	-8.2±5.1	-8.9±5.1	-7.7±6.3	-8.9±6.8	0.754
Knee flexion angle at IC	27.1±7.6	26.3±7.1	26.7±5.6	24.8±8.7	0.651
Knee min flexion angle at ST	16.0±7	13.6±8.0	11.0±6.9	11.8±5.9	0.090

Knee max flexion angle at SW	55.4±7.8	59.4±8.5	55.4±10.4	60.3±9.0	0.720
Ankle DF angle at IC	0.7±4.6	-1.5±6.2	1.1±4.3	-2.8±6.6	0.432
Ankle max DF angle at ST	15.6±4.0	14.2±3.7	15.4±5.4	14.5±4.2	0.693
Ankle max PF at SW	-4.9±5.6	-9.4±7.1	-6.8±6.5	-11.6±6.6	0.873

Data presented as mean ± standard deviation; * $P < 0.05$ was statistically significant for time × intervention interaction.; ST, stance phase; IC, initial contact; SW, swing phase; DF, dorsiflexion; PF, plantarflexion;

Supplementary Table S3. Comparison of Kinetic Parameters

	**AP training group**		**Control group**		
	**Before training**	**After training**	**Before training**	**After training**	**P value**
**Affected side**					
Hip max flexion moment	0.4±0.2	0.3±0.1	0.3±0.2	0.3±0.1	0.512
Hip max extension moment	-0.3±0.1	-0.3±0.	-0.3±0.1	-0.4±0.2	0.260
Hip max power generation	0.8±0.3	0.8±0.4	0.7±0.2	0.8±0.3	0.794
Hip max power absolution	-0.9±0.8	-0.8±0.6	-0.7±0.7	-0.6±0.7	0.945
Knee max flexion moment	0.3±0.2	0.3±0.2	0.2±0.2	0.2±0.3	0.975
Knee max extension moment	-0.2±0.1	-0.2±0.1	-0.2±0.1	-0.1±0.1	0.991
Knee max power generation	1.8±1.2	1.8±0.9	1.6±1.0	1.7±1.0	0.736
Knee max power absolution	-0.3±0.1	-0.3±0.1	-0.3±0.1	-0.2±0.1	0.612
Ankle max PF moment	0.6±0.3	0.6±0.2	0.6±0.2	0.6±0.3	0.907
Ankle max DF moment	-0.0±0.0	-0.0±0.0	-0.0±0.1	-0.0±0.1	0.980
Ankle max power generation	9.2±0.7	9.1±1.3	9.2±0.8	9.3±0.8	0.554
Ankle max power absolution	-0.1±0.0	-0.1±0.0	-0.1±0.0	-0.1±0.0	0.816
**Unffected side**					
Hip max flexion moment	0.5±0.2	0.5±0.2	0.5±0.2	0.4±0.1	0.560
Hip max extension moment	-0.5±0.2	-0.5±0.2	-0.4±0.2	-0.5±0.3	0.840
Hip max power generation	0.6±0.2	0.8±0.2	0.7±0.3	0.7±0.3	0.256
Hip max power absolution	-1.6±0.6	-1.7±0.4	-1.4±0.5	-1.5±0.5	0.766
Knee max flexion moment	0.7±0.2	0.7±0.2	0.6±0.2	0.6±0.3	0.948
Knee max extension moment	-0.2±0.0	-0.2±0.1	-0.2±0.1	-0.2±0.0	0.407
Knee max power generation	3.1±0.7	3.1±0.5	2.7±0.8	2.9±0.8	0.366
Knee max power absolution	-0.7±0.2	-0.7±0.2	-0.6±0.2	-0.6±0.2	0.895
Ankle max PF moment	0.8±0.2	0.8±0.2	0.7±0.2	0.8±0.2	0.246
Ankle max DF moment	-0.0±0.0	-0.0±0.0	-0.0±0.0	-0.0±0.1	0.511
Ankle max power generation	9.6±0.8	9.7±0.7	9.4±0.5	9.3±0.7	0.612
Ankle max power absolution	-0.1±0.1	-0.2±0.1	-0.2±0.1	-0.1±0.2	0.082

Data presented as mean ± standard deviation; * $P < 0.05$ was statistically significant for time × intervention interaction.; PF, plantarflexion; DF, dorsiflexion;
